# Key factors of the functional ability of older people to self-manage medications

**DOI:** 10.1038/s41598-021-01434-9

**Published:** 2021-11-12

**Authors:** Ana Margarida Advinha, Carla Nunes, Carla Teixeira de Barros, Manuel José Lopes, Sofia de Oliveira-Martins

**Affiliations:** 1 CHRC – Comprehensive Health Research Centre, Lisbon, Portugal; 2 Universidade de Évora, Évora, Portugal; 3 Escola Nacional de Saúde Pública, Universidade Nova de Lisboa, Lisbon, Portugal; 4 Faculdade de Farmácia, Universidade de Lisboa, Lisbon, Portugal

**Keywords:** Geriatrics, Drug therapy

## Abstract

Daily medication use can be affected by the gradual loss of functional ability. Thus, elderly patients are at risk for nonadherence due to functional decline, namely, decreases in cognitive skills and visual and manual dexterity. The main objective was to assess the ability of older people to self-manage their medication and to identify the main predictors for unintentional nonadherence. A cross-sectional study was conducted (2014–2017) in community centers and pharmacies. Functional assessment was performed with the Portuguese versions of the Drug Regimen Unassisted Grading Scale (DRUGS-PT) and the Self-Medication Assessment Tool (SMAT-PT). A purposive sample including 207 elderly patients was obtained. To identify the main predictors, binary logistic regression was performed. The average DRUGS-PT score was slightly lower than that in other studies. On the SMAT-PT, the greatest challenge for patients was identifying medications by reading labels/prescriptions. The main difficulties identified were medication memorization and correct schedule identification. The scores were higher with the real regimen than with the simulated regimen, underlining the difficulties for patients in receiving new information. Regarding the predictors of an older individual’s ability to self-manage medications, two explanatory models were obtained, with very high areas under the curve (> 90%). The main predictors identified were cognitive ability, level of schooling and daily medication consumption.

## Introduction

Adherence to a medication regimen implies the desire and the ability to take the medicine as it has been prescribed. In fact, adhering to the daily therapeutic regimen for a medication can become a complex process and can be affected by systematic errors, namely, due to a gradual loss of the functional ability to manage medications^1–3^. Thus, Maddigan (2003) defined the functional ability to self-manage medications as the cognitive and physical capacity to self-administer/take medication according to the prescription received^4^.

Decreases in cognitive skills, visual acuity and manual dexterity have significant impacts on unintentional nonadherence, which may lead to health problems as a result of missed or incorrect medication administration^1, 3–7^. In this context, older patients represent a risk group for nonadherence due to their diminished functional ability. According to the World Health Organization (WHO), approximately 46% of individuals aged 60 years or older have some type of disability. The main causes observed were related to visual acuity, dementia, hearing loss and osteoarthritis^8^. These causes were also confirmed by the latest data from the *Global Burden of Disease*, where musculoskeletal, mental/neurological and sense organ diseases are primarily responsible for older people living with a disability for years^9^.

The use of medications for older patients presents many advantages and is certainly one of the main factors that contributes to the increase in life expectancy, but there are also some associated risks. A lack of the functional ability to manage medications or adhere to the prescribed regimens are possible sources of medication-related problems that can be avoided or minimized. In this daily activity, attention should be given to the multiple problems that can result from an older person’s lack of physical and cognitive skills to take medications^3, 5, 10–16^.

Although the ability to manage medications may be reduced, it is known that performance can be enhanced, namely, if interpreted through the self-care deficit theory, as a task that the individual develops with a goal to improve or maintain their own health^6, 17, 18^. According to this approach, the performance of older people in managing their own medications can be improved with the introduction of support for this daily activity, whether it be human support (e.g., caregivers or health professionals), physical support (e.g., pillboxes) or digital devices (e.g., digital apps)^4, 19^.

To understand the main barriers and requirements of older people regarding medication self-management, it is essential to assess functional capacity using specific tools, especially with the aim of identifying previous difficulties in community-dwelling older individuals^1, 3, 20^.

In addition, it is also important to identify the factors that allow for the prediction of the loss of the functional ability to manage medications, which, in fact, has not yet been robustly explored in previous studies.

The main objectives of this study were (1) to assess the functional ability of community-dwelling older individuals to manage their own medications using the Drug Regimen Unassisted Grading Scale, Portuguese version (DRUGS-PT), and the Self-Medication Assessment Tool, Portuguese version (SMAT-PT), two tools that have been adapted to and validated in the Portuguese elderly population; and (2) to explore the predictors of older people’s functional ability to manage medications, based on the evidence found in a previous systematic literature review regarding relevant dimensions that are potentially related to medication management ability^21^.

## Methods

### Study design, setting and participants

A cross-sectional design was implemented in day centers for community-based older people and community pharmacies in the Alentejo Region, Portugal (data collection from 2014 to 2015, tool validation in 2016 and study of predictors in 2017). Purposive sampling was adopted based on older people who were previously interviewed for the adaptation process and a validation study^22, 23^. All older people who met the inclusion criteria—native Portuguese-speaking, community-dwelling residents of the Alentejo Region who were over 65 years old, taking at least one chronic medication and had no visible signs of dementia—were invited to participate in the study; those who accepted were included. Eligible older individuals were consecutively recruited, informed about the objectives of the study, and signed an informed consent form at the time of their first visit. Consenting participants answered all the questions in the interviews, including questions about the main measures of medication adherence, medication regimen complexity, cognitive status, and functional capacity for medication management. To maximize the sensitivity, the sample size was calculated based on the general ratio of ten subjects for each evaluated subject^24–26^. A sample of 207 older individuals was interviewed by a pharmacist who was qualified and trained to administer all measurement tools.

### Measurements

The assessment of older people’s functional ability to manage their own medications, with the specific goal of identifying and analyzing the main predictors for this basic daily activity, was performed by the authors with the following previously validated tools, both of which are already published and where specific application details could be found: the DRUGS-PT^22^ and SMAT-PT^23^.

#### Functional capacity for medication management

The DRUGS-PT and SMAT-PT were developed to assess the ability of older people to manage their own medications. As measures of the capacity for medication self-management, both tools were based on four tasks for each individual medicine in the real medication regimen that the subject was taking at the time of the assessment. The tasks were performed by the subjects, but the form was filled out by the interviewer. The questionnaire covered the daily habits and mealtimes in relation to the medication-taking schedule and asked the subjects about each task related to medication self-management: the identification, access (opening the package), dosage and timing. Each subject’s response was recorded as either able (1 point) or unable (0 points).

Other measures were applied, with the objective of assessing the dimensions potentially related to medication management ability.

#### Medication adherence

It was assessed with the Morisky Medication Adherence Scale (MMAS)^27^, adapted in Portuguese as the MAT (therapeutic adherence measure)^28^.

#### Medication regimen complexity

It was calculated according to the Medication Regimen Complexity Index (MRCI)^12^, adapted in Portuguese by Melchiors et al.^29^.

#### Cognitive status

It was assessed in two phases: first, with the Mini-Mental State Examination (MMSE) to identify cognitive impairment^30, 31^; and second, with the Clock Drawing Test (CDT) to evaluate the individual’s ability to read, understand and write times of day. In this case, a global clock test was performed and divided into three subtests using the tools developed by Atalaia-Silva et al.^32^, Lam et al.^33^, Freedman et al.^34^ and Tuokko et al.^35^.

#### Instrumental activities of daily living (IADL)

IADL were classified with the Lawton scale^36^, adapted in Portuguese by Botelho^37^.

The information about the medications taken by the participants, which also served as the basis for the application of the MRCI and the assessment of the older person’s ability to manage their own medications with the DRUGS-PT and SMAT-PT, was obtained through self-reports and double-checked with pharmacy/medical records and medication bags.

To identify the ability to self-manage medications, two explanatory models were developed: the first model, with the cutoff point set at the median (0.9) to define high (> 0.9) and low (< 0.9) ability to manage medications (Binary I); and the second model, where high ability was defined as the full ability to manage medications with no errors (high ability = 1.0 and low ability < 1.0; Binary II).

### Statistical analysis

A descriptive analysis was performed for the applicable variables (demographic and clinical variables). The Kolmogorov–Smirnov test was applied to test the normality of the data distribution, the Spearman test was used to evaluate correlations between the variables, the Wilcoxon-Mann–Whitney and Kruskal–Wallis tests were used to compare the functions of variable distribution, and the chi-square test was used to compare the variables between the nominal and categorical variables from independent samples.

The study of older people’s medication management ability predictors was performed using binary logistic regression, and according to the binarization of the dependent variable, it was ranked as high and low, using the median.

In the regression analysis, a step-forward approach was used, verifying the quality of the data with the Hosmer–Lemeshow test. ROC curves were calculated to obtain the sensitivity and specificity of the different models. Statistical analysis was conducted using SPSS Statistics, IBM (Version 22), and a significance level of 5% was adopted (95% confidence interval).

### Ethics statement

This study was performed according to the ethical standards of the Declaration of Helsinki. The study protocol was reviewed and approved by the Ethics Committee of the University of Évora. The protocol was fully implemented according to the protocol approved by the Ethics Committee.

### Ethics approval

University of Évora Ethics Committee Approval, Document Number 14009.

### Consent to participate/publication

All participants were informed about the objectives of the study and signed free and informed consent forms.

## Results

### Social and demographic features

In the sample (*n* = 207), most individuals were female (75.4%). The subjects’ ages ranged between 65 and 93 years, with an average of 75.5 (SD = 6.6) years (75.4 (SD = 6.8) years in women and 76.0 (SD = 5.8) years in men). The sample was distributed among the four subregions of Alentejo and comprised users of day centers for older people and community pharmacies. Most participants were married (51.2%), and a large minority were widowed or single (44%). Their households were made up of an average of 1.8 (SD = 0.8) people, with the majority being made up of two people (47.8%); 36.2% of the participants lived alone.

Only 19.8% of the participants reported that they did not know how to read/write, although 23.2% of them had never attended school. Of those who had gone to school, the majority (61.8%) took classes for one to four years. The income of 98.1% of the interviewees came from retirement pensions (yields considered low), with approximately 37.2% of the interviewees reporting financial difficulties in acquiring medications. The most frequently mentioned option for purchasing medicines was to pay the pharmacy at the end of the month (12.6%). However, 6.8% of the participants reported having chosen not to buy their prescribed medications due to financial difficulties. In some cases (2.4%), the subjects reported that they had stopped buying other essential products or services so that they could afford their medications. Approximately 47.8% of the sample considered their monthly expenses (with medication) to be high in relation to their income.

### Health conditions

Regarding the perception of their own health status, 54.6% of the sample considered their health to be reasonably good. Compared with their peers, 43% of the individuals considered themselves to be in good health.

In cognitive terms, the sample obtained an average MMSE score of 25.7 points (SD = 3.8) [13.0–30.0]. In general, the older subjects had the greatest difficulties with the most complex components, namely, the reproduction of images (46.4% of the total disabilities) and written information (41.6% of the total disabilities). It was also found that among older people, the younger age group (65–69 years) (*p* = 0.02), as well as those who attended school for a longer period (*i.e.*, 1–9 years of school) (*p* < 0.001), had significantly higher cognitive ability than the rest of the sample. Regarding the Clock Drawing Test (CDT), which tests a subject’s ability to draw a clock (average score 7.8 (SD = 4.1) [0.0–13.0]) and to indicate (average score 25.7 (SD = 19.4) [0.0–55.0]) and read the time (average score 8.1 (SD = 4.1) [0.0–15.0]), men slightly outperformed women, specifically in the exercises of time indication (*p* = 0.02) and reading (*p* = 0.02). Age also had a statistically significant effect on the sample’s CDT performance. Additionally, in the CDT, older individuals between 65 and 69 years of age and older individuals with higher levels of education showed greater cognitive capacity than individuals in the other groups (*p* < 0.001).

Finally, in terms of IADL, the sample had an average score of 19.1 (SD = 4.4) [2.0–24.0]. The tasks in which the elderly patients reported the greatest functional dependence were the use of transportation (54.1%), shopping (39.1%) and preparing meals (22.7%). Conversely, the tasks in which the older patients showed the greatest functional independence were using the telephone (97.6%), managing money (97.1%) and taking medications (90.8%).

### Medication profiles

All the older individuals included in this study (*n* = 207) took at least one daily medication on a chronic basis, with an average of 6.7 (SD = 3.3) medications per day [1.0–24.0]. Women (mean 7.1 (SD = 3.6)) had a significantly higher daily medication consumption than men (mean 5.7 (SD = 2.4)) (*p* = 0.014). In terms of age groups, there were no statistically significant differences. The health conditions for which the sample took the most medications involved the cardiovascular system (36.9%), the nervous system (19.4%) and, tied for third place, the gastrointestinal and metabolic systems (16.4%). The most used active substances were low-dose acetylsalicylic acid (4.5%), simvastatin (4.3%) and atorvastatin (2.9%).

In the self-reports of medication adherence, the sample had an average global score of 5.8 (SD = 0.6) [3.0–6.0]. When a more refined analysis of this measure was performed, two aspects could be observed: (1) when adherence was analyzed based on the 50th-percentile value (median = 6.0), it was found that 81.2% of the individuals were adherent; and (2) when adherence was defined as the maximum score (score = 6.0) on all items of the scale, it was observed that only 9.2% of the sample was fully adherent.

The therapeutic complexity of the sample, measured using the MRCI, had an average score of 15.7 (SD = 9.6) [2.0–59.5]; median = 4.0). There were statistically significant differences in the total MRCI scores between the sexes (*p* = 0.01), with higher scores in women. In cases where the number of daily medications taken was higher, the therapeutic complexity was greater (*p* < 0.001).

### Medication self-management ability

Medication self-management ability was assessed by the DRUGS-PT and the SMAT-PT. For the DRUGS-PT, the average score was 80.7 (SD = 25.7) [2.9–100.0]. The main difficulties identified were the identification of the medication, the opening of the package and medication dose scheduling.

The problem with the identification of medications was mainly related to the drug names. Most of the participants had difficulty saying or memorizing the names for multiple reasons (e.g., foreign languages, extensive designations, or similarity to other names). This fact emphasizes the use of alternative options, such as physical characteristics (e.g., the color or shape of the package/dosage form), written indications on the package/label, or pharmaceutical pictograms. However, these alternative options encounter barriers, such as the similarities between generic drugs (e.g., the majority are round white pills) and the frequent changes in the drug packages/labels.

When the evaluation was made with the SMAT-PT, two different medication regimens were used—simulated and real. The SMAT-PT was divided into five scales: functional ability and cognitive ability (using a simulated regimen), medication memorization, self-reported adherence, and intentional nonadherence (using the real regimen). For functional and cognitive abilities, the average scores were 20.6 (SD = 7.0) [6.8–28.0] and 38.4 (SD = 6.6) [2.0–44.0], respectively. For the real regimen, the average scores obtained were 83.6 (SD = 15.6) [6.8–100.0] for medication memorization, 97.2 (SD = 11.0) [0.0–100.0] for self-reported adherence and 4.9 (SD = 10.1) [0.0–64.4] for intentional nonadherence. With the simulated regimen, the greatest challenge for the sample was identifying medication by reading labels/prescriptions. Some difficulties were identified in distinguishing colors, specifically dark colors. In the cognitive dimension, the main difficulty was the identification of daily medication schedules. With the real regimen, the greatest difficulties for the sample were memorizing medications, especially identifying medications by name.

There was a direct association between education level, cognitive ability, and the ability to manage medications.

### Predictors of medication self-management ability

As described in the methods, to identify predictors of older people’s functional ability to manage their medications, two explanatory models of the dependent variable were developed, both of which presented excellent discriminant capacity. To identify the covariables with statistically significant effects, each binary dependent variable was entered in a logistic regression model (Table 1).

**Table 1:** Logistic regression models.

Independent variables	Binary dependent variable I (high ability > 0.9 and low ability < 0.9)						Binary dependent variable II (high ability = 1.0 and low ability < 1.0)					
	(1) Unadjusted values			(2) Adjusted values *^a^			(1) Unadjusted values			(2) Adjusted values *^a^		
	OR	CI 95%	*p*	OR*	CI 95%	*p*	OR	CI 95%	*p*	OR*	CI 95%	*p*
**Sex**												
**♀**	1	–	–				1	–	–			
**♂**	1.13	0.60–2.12	0.72				0.78	0.39–1.56	0.48			
**Age**^b^ **(years)**												
65–69	1	–	–				1	–	–			
70–74	1.96	0.81–4.78	0.14				2.14	0.91–5.06	0.08			
75–79	5.67	2.44–13.14	< 0.001				3.85	1.66–8.91	0.002			
80–84	3.37	1.32–8.58	0.01				6.00	1.98–18.19	0.002			
≥ 85	7.93	2.35–26.73	0.001				8.50	1.76–41.06	0.01			
**Education level**^c^ **(years)**												
0	1	–	–	1	–	–	1	–	–	1	–	–
1–4	0.10	0.04–0.26	< 0.001	0.13	0.05–0.33	< 0.001	0.09	0.02–0.38	0.001	0.11	0.03–0.49	0.004
≥ 5	0.04	0.01–0.13	< 0.001	0.04	0.01–0.16	< 0.001	0.05	0.01–0.24	< 0.001	0.06	0.01–0.31	0.001
**MMSE**	0.70	0.62–0.79	< 0.001	0.72	0.63–0.81	< 0.001	0.71	0,61–0.83	< 0.001	0.73	0.62–0.86	< 0.001
**CDT**^d^												
CDT-1	0.73	0.66–0.81	< 0.001	0.74	0.67–0.82	< 0.001	0.77	0.69–0.86	< 0.001	0.81	0.72–0.90	< 0.001
CDT-2	0.94	0.93–0.96	< 0.001	0.94	0.93–0.96	< 0.001	0.95	0.94–0.97	< 0.001	0.96	0.94–0.98	< 0.001
CDT-3	0.76	0.70–0.83	< 0.001	0.77	0.70–0.85	< 0.001	0.79	0.72–0.87	< 0.001	0.83	0.75–0.92	< 0.001
Total CDT	0.95	0.94–0.96	< 0.001	0.95	0.93–0.97	< 0.001	0.96	0.95–0.97	< 0.001	0.96	0.95–0.98	< 0.001
**IADL**	0.92	0.86–0.98	0.01	0.95	0.88–1.03	0.20	0.91	0.84–0.99	0.02	0.94	0.85–1.03	0.19
**Daily medication consumption**^e^	1.05	0.96–1.14	0.28	1.03	0.94–1.12	0.58	1.23	1.10–1.39	0.001	1.21	1.07–1.36	0.003
**MAT**	0.97	0.88–1.07	0.50	0.95	0.86–1.06	0.36	0.98	0.88–1.10	0.76	0.98	0.87–1.10	0.72
**MRCI**^f^												
A	0.99	0.88–1.11	0.84	0.99	0.88–1.12	0.87	1.09	0.94–1.27	0.24	1.09	0.93–1.28	0.27
B	1.02	0.96–1.08	0.55	1.01	0.95–1.07	0.86	1.14	1.05–1.24	0.002	1.13	1.03–1.23	0.01
C	0.99	0.93–1.07	0.92	0.99	0.92–1.07	0.79	1.10	1.00–1.20	0.05	1.09	0.99–1.20	0.08
Total	1.00	0.98–1.03	0.83	1.00	0.97–1.03	0.96	1.06	1.02–1.11	0.01	1.06	1.01–1.10	0.02

^a^Sex- and age-adjusted values.

^b^Age reclassification.

^c^Education level reclassifications (years).

^d^Clock Drawing Test (CDT): CDT-1 (clock drawing), CDT-2 (time indication) and CDT-3 (hour reading).

^e^Daily medication consumption corresponds to the number of regular medications.

^f^Medication Regimen Complexity Index (MRCI): Section A (dosage form), Section B (dosage frequency) and Section C (additional instructions).

According to the covariables that were previously studied, new adjusted models were generated from the Binary I and II dependent variables using the *forward:LR method*. The model quality and validity were verified by a waste analysis and a Hosmer–Lemeshow test (*p* > 0.05). In the final models, fourteen records were excluded from Binary model I, corresponding to two missing values and twelve waste values, and sixteen records were excluded from Binary model II, corresponding to two missing values and fourteen waste values.

The observations and predictions for each class of binary variables are presented in Table 2.

**Table 2:** Observations and predictions of the Binary I and Binary II models.

**Binary I model**				
	Predictions			
Observations	High ability (> 0.90)	Low ability (< 0.90)	Total	% of correct classifications
High ability (> 0.90)	82	19	101	81.20
Low ability (< 0.90)	22	70	92	76.10
Total			193	

**Binary II model**				
	Predictions			
Observations	High ability (= 1.00)	Low ability (< 1.00)	Total	% of correct classifications
High ability (= 1.00)	26	18	44	59.10
Low ability (< 1.00)	11	136	147	92.52
Total			191	

The new models for each binary dependent variable showed the following:

1. *Binary I model* education level (*p* ≤ 0.04), cognitive status expressed by the MMSE (*p* = 0.001) and cognitive status expressed by the CDT (*p* < 0.001) presented statistically significant effects (Table 3).
  The probability that an older individual would be able to manage medications, given a cutoff point of 0.9, increased exponentially with education level (30% for 1–4 years of schooling and 10% for 5 years of schooling) and cognitive ability (75% with the MMSE and 94% with the CDT). This model presented a sensitivity (capacity to correctly identify high-ability cases) of 81.2% and a specificity (capacity to correctly identify low-ability cases) of 76.1%. The discriminative capacity was exceptional, with an ROC of 0.908 (*p* < 0.001).
2. *Binary II model* cognitive status expressed by the MMSE (*p* = 0.002), cognitive status expressed by the CDT (*p* < 0.001) and daily medication consumption (*p* < 0.001) had statistically significant effects (Table 4).

**Table 3:** Independent variables in the new binary I model.

Independent variables		Entries	OR	CI 95%	*p*
**Education level (years)**	0	Step 3/3	1	–	0.02
	1–4		0.30	0.09–0.94	0.04
	≥ 5		0.10	0.02–0.52	0.01
MMSE		Step 2/3	0.75	0.63–0.90	0.001
Total CDT		Step 1/3	0.94	0.92–0.96	< 0.001

**Table 4:** Independent variables in the new binary II model.

Independent variables	Entries	OR	CI 95%	*p*
MMSE	Step 3/3	0.67	0.51–0.86	0.002
Daily medication consumption	Step 2/3	1.54	1.26–1.89	< 0.001
Total CDT	Step 1/3	0.95	0.93–0.98	< 0.001

The probability that an older individual would be able to manage medications with total accuracy (100%) increases exponentially with cognitive competence (67% with the MMSE and 95% with the CDT). A similar result was observed for daily medication consumption, where the probability of an older person being able to manage medications increased 54% for each medication taken. This model presented a sensitivity of 59.1% and a specificity of 92.5%. The discriminative capacity was exceptional, with an ROC of 0.899 (*p* < 0.001).

## Discussion

The main goal of this study was to identify and analyze the main predictors for the medication management ability of older people using previously validated tools to assess the functional ability of older people to self-manage their medication—the DRUGS-PT^22^ and the SMAT-PT^23^.

However, based on the literature review, other measures were applied with the objective of assessing dimensions potentially related to medication management ability: medication adherence, medication regimen complexity, cognitive status, and other activities of daily living.

In the sampling procedure, although it was a documented practice used in some similar studies^2, 15, 38–49^, it was decided not to use cognitive status as an exclusion criterion because those tests, especially the MMSE, were not administered before the interview took place. Thus, a cutoff value was not established for older individuals to be excluded/included according to a previous cognitive assessment, since it was necessary to understand how older people, without apparent signs of dementia or severe cognitive impairment, manage their medication in a community environment, especially in their own homes. It should be stated that cognitive impairment occurs naturally over the years, as previously mentioned by Park et al.^50^, and does not necessarily represent a pathological condition.

The DRUGS–PT used the real medication regimen taken by the patient. The average score obtained on the DRUGS-PT, although similar to those found in a systematic literature review of other studies that used the same instrument^15, 43–45, 47, 51^, was slightly lower in this study, which can be explained by the fact that older people with mild to moderate cognitive impairment were not excluded, a methodological choice that we have justified above.

Two different medication regimens—simulated and real—were used for the SMAT-PT. Regarding the functional and cognitive ability of older people to manage the simulated regimen (standard), the greatest difficulties arose in identifying medications by reading the labels and prescriptions. In the scales based on the real regimen—medication memorization, self-reported adherence, and intentional nonadherence—the greatest difficulties were in memorizing medications, especially in identifying medications by name, an area in which scores were especially low. There was some difficulty concerning the cognitive component, especially in identifying the periods of the day in which medication should be taken (schedules). Even though the older subjects easily understood information about the prescribed doses, the articulation of the dose with the administration timing was not clear. It was reported that the use of daily routines would support accurate medication use (31% of the participants) (e.g., weekly pillboxes, annotated medication labels and association of medication intake with meals). On the SMAT-PT, the scores obtained with the real regimen were better than the scores obtained with the simulated regimen, indicating the complications that have already been mentioned by other authors regarding treatment and the apprehension of new information^1, 3, 52, 53^.

The main predictors found for medication management capacity were the cognitive measures, the MMSE and the CDT (for either cutoff point) scores, the level of schooling (given the median as the cutoff point) and the daily consumption of drugs (given a criterion of total accuracy). Maddigan (2003) previously identified the effects of cognition and medication regimen complexity as important predictors of medication management capacity^4^. The level of schooling identified in the results could be explained by the low level of literacy of older Portuguese people, which could influence all behaviors, namely, the capacity to manage medications.

The main predictors of the ability to self-manage medications were found with precision, especially for cognitive status, which, in terms of public health interventions, will allow for the easy identification of patients who may need intervention. The identification of predictors of the ability to manage medication in older adults, such as cognitive status, seems to be an important finding that will allow for the designs of more effective interventions, as previous studies were unable to determine the impact of interventions on medication‐taking ability (educational and behavioral interventions)^54^.

Applicability testing of validated instruments for Portugal in different community contexts, especially in the home and in primary health care clinics, as well as in global geriatric assessments, should also be considered.

From this point of view, the present work should be recognized as important for the conduct of new research and the development of proposals for community interventions since medication management per se plays a fundamental role in the health and quality of life of older people and has been explored very little to date.

### Limitations

Some limitations of the study were identified. The first is the fact that the sample was a purposive sample recruited in two different settings (community pharmacies and day centers). Although we do not think that it calls the obtained results into question, a larger sample could make the results more robust. Considering this, the ROC curves obtained, with very high values, in the step-by-step regression analysis do not indicate limitations caused by the sample dimension. However, the potential for selection bias must also be considered, as well as other biases of population representativeness that may limit the extrapolation of the results to the general population.

Regarding the data used for the identification of predictors (regression), the fact that some of the instruments were based on self-reported data is a limitation (for example, self-reported adherence (subjective) and self-reported medication use, rather than objective measures (e.g., pill count, dispensing records). However, the implementation of the study was already difficult, and self-reported measures have been proven, in several studies, not to negatively influence the results. In some situations, self-reported medication use, rather than prescription records, could be more sensitive, as it relates to the real medications that the individual is taking^55^.

## Conclusion

The functional ability of older people to self-manage medications was found to be clearly associated with cognitive impairment. These findings underscore the benefit of alerting researchers/health care professionals to the importance of these evaluations in the context of global geriatric assessment and encourage the use of specific tools. Different stakeholders in the care of older people should be involved in this process of evaluating and supporting medication management. Interoperability between services should be pursued so that the process can achieve positive and integrated results for the health of older people, promoting their ability to live independent, healthy lives in their own homes for the longest possible period.

## Data Availability

The database and analyses for the present study are available through the corresponding author upon reasonable request.
